# Supplementary material for: Genome-Wide Investigation and Characterization of SWEET Gene Family with Focus on Their Evolution and Expression during Hormone and Abiotic Stress Response in Maize
Source: Genes (Basel). 2022 Sep 20;13(10):1682. doi: 10.3390/genes13101682 (PMC9601529; doi:10.3390/genes13101682)
Supplement: Supplementary file 1 [file genes-13-01682-s001.zip › Supplementary Figures 1 and 2.pdf]

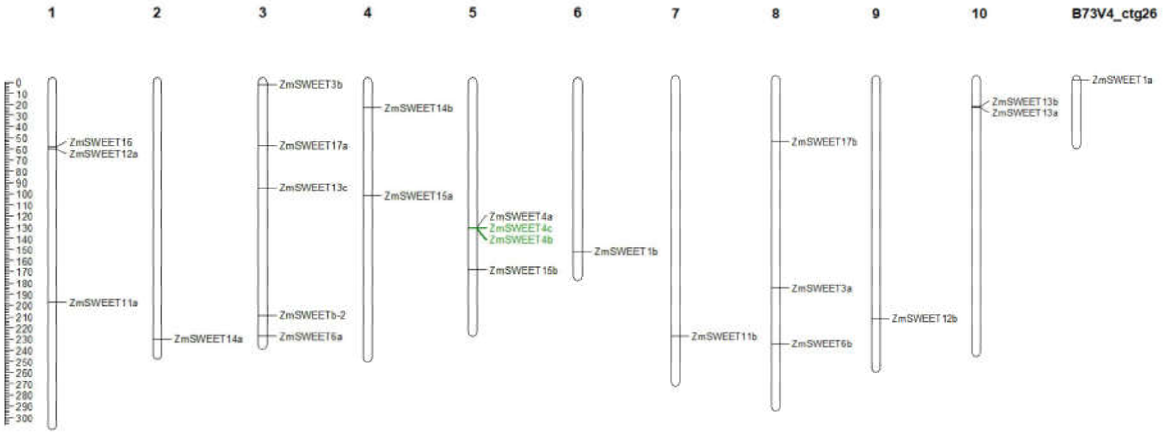

Figure S1. Chromosomal distribution of ZmSWEET genes. Tandem repeat genes are labeled in green.

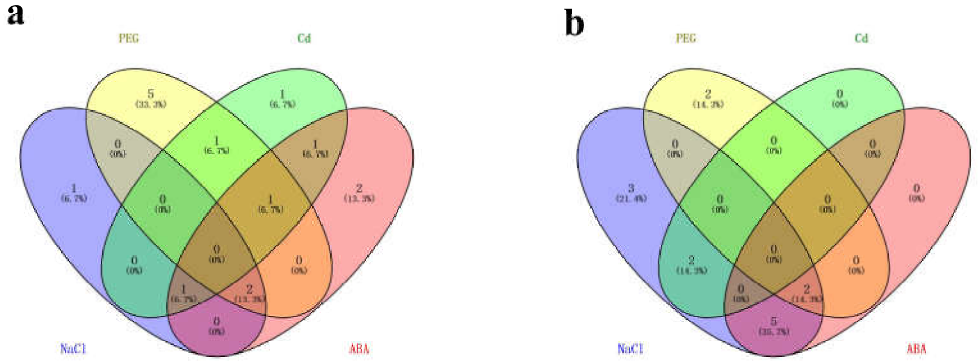

Figure S2. Venny diagram of the ZmSWEET relative expression under four treatments. a The number of ZmSWEET gene is induced under four treatments. b The number of ZmSWEET gene is inhibited under four treatments. Blue oval-shaped, yellow oval-shaped, green oval-shaped and red oval-shaped indicate NaCl treatment, PEG treatment, Cd treatment and ABA treatment, respectively.
