# Supplementary material for: Genome-Wide Investigation and Characterization of SWEET Gene Family with Focus on Their Evolution and Expression during Hormone and Abiotic Stress Response in Maize
Source: Genes (Basel). 2022 Sep 20;13(10):1682. doi: 10.3390/genes13101682 (PMC9601529; doi:10.3390/genes13101682)
Supplement: Supplementary file 1 [file genes-13-01682-s001.zip › Table S1.pdf]

Table S1. Interchromosomal relationships of SWEET genes in maize, foxtail millet, rice and sorghum, respectively.

Type	Duplicated Gene Pairs	Ka	Ks	Ka/Ks	Duplicated type	Purify Selectio n	T(Mya)
Zea mays	ZmSWEET4b&ZmSWEET4c	0.0978233	0.72822	0.134332	TD	YES	56.01692308
Zea mays	ZmSWEET6a&ZmSWEET6b	0.0146144	1.08406	0.0134813	WGD or SD	YES	83.38923077
Zea mays	ZmSWEET11a&ZmSWEET11b	0.955285	1.11518	0.85662	WGD or SD	YES	85.78307692
Zea mays	ZmSWEET12a&ZmSWEET12b	0.0919998	0.392585	0.234344	WGD or SD	YES	30.19884615
Zea mays	ZmSWEET13c&ZmSWEET13b	0.0570968	0.544807	0.104802	WGD or SD	YES	41.90823077
Zea mays	ZmSWEET17a&ZmSWEET17b	0.0509797	0.292778	0.174124	WGD or SD	YES	22.52138462
Setaria italica	KQL02677&KQL25234	0.232695	1.1622	0.200219	WGD or SD	YES	89.4
Setaria italica	KQL07993&KQL15405	0.189161	1.07293	0.176303	WGD or SD	YES	82.53307692
Setaria italica	KQL05781&KQL14056	1.00143	0.99597	1.00549	WGD or SD	NO	76.61307692
Setaria italica	KQK94690&KQK94691	0.0712048	1.00087	0.0711432	TD	YES	76.99
Setaria italica	KQL29317&KQL29318	0.034184	0.629107	0.0543374	TD	YES	48.39284615
Setaria italica	KQL16417&KQL16419	0.0960148	0.612411	0.156782	TD	YES	47.10853846
Setaria italica	KQL13516&KQL13517	0.152133	0.455383	0.334076	TD	YES	35.02946154
Oryza sativ	Os01t0605700-01&Os05t0588500-01	0.204063	3.89706	0.0523632	WGD or SD	YES	299.7738462
Oryza sativ	Os01t0220700-00&Os05t0214300-00	0.33398	1.36615	0.244468	WGD or SD	YES	105.0884615
Oryza sativ	Os08t0535200-01&Os09t0508250-00	0.324364	6.23561	0.0520179	WGD or SD	YES	479.6623077
Oryza sativ	Os01t0605700-01&Os01t0606000-00	0.0211796	0.655377	0.0323167	TD	YES	50.41361538
Sorghum bicolor	EES01039&EES20039	0.298998	0.490543	0.609525	WGD or SD	YES	37.73407692
Sorghum bicolor	EES14183&OQU89754	0.181028	0.81962	0.220869	WGD or SD	YES	63.04769231
Sorghum bicolor	EES19554&KXG33819	0.135061	1.26622	0.106665	WGD or SD	YES	97.40153846
Sorghum bicolor	OQU79094&EES15957	0.0390812	0.261587	0.1494	TD	YES	20.12207692
