# Supplementary material for: Genome-Wide Investigation and Characterization of SWEET Gene Family with Focus on Their Evolution and Expression during Hormone and Abiotic Stress Response in Maize
Source: Genes (Basel). 2022 Sep 20;13(10):1682. doi: 10.3390/genes13101682 (PMC9601529; doi:10.3390/genes13101682)
Supplement: Supplementary file 1 [file genes-13-01682-s001.zip › Table S4.pdf]

Table S4. The identification of SWEET members in rice, foxtail millet and sorghum

Gene ID	Transcript ID	length	MW(Da)	pI
Os01t0605700	Os01t0605700-01	254	27955.4	9.84
Os05t0426000	Os05t0426000-01	261	27621.9	8.35
Os12t0476200	Os12t0476200-01	296	31924.7	9.18
Os05t0588500	Os05t0588500-01	237	25916.6	8.17
Os01t0220700	Os01t0220700-00	130	14777.1	8.48
Os01t0700100	Os01t0700100-01	230	25477	8.91
Os03t0341300	Os03t0341300-01	254	27327.2	8.49
Os02t0513100	Os02t0513100-01	319	34964.2	7.44
Os11t0508600	Os11t0508600-01	303	32700.3	8.7
Os01t0881300	Os01t0881300-02	273	29502.5	8.46
Os02t0301100	Os02t0301100-01	259	28046.1	9.59
Os09t0258700	Os09t0258700-00	222	24840.5	9.69
Os01t0314600	Os01t0314600-00	156	17227.4	8.49
Os08t0535200	Os08t0535200-01	307	32977.8	9.54
Os05t0214300	Os05t0214300-00	260	29481	9.16
Os12t0178500	Os12t0178500-00	209	23019.3	9
Os01t0541800	Os01t0541800-01	243	26746.5	8.81
Os03t0347500	Os03t0347500-01	300	32512.1	5.26
Os09t0259200	Os09t0259200-00	180	20224.3	10.71
Os01t0606000	Os01t0606000-00	259	28169.5	9.34
Os09t0256650	Os09t0256650-00	98	11096.3	9.54
SETIT_018169mg	KQL29318	251	27390.5	9.54
SETIT_019403mg	KQL29315	315	34633.7	10.04
SETIT_017821mg	KQL29789	319	34438.7	7.87
SETIT_019459mg	KQL29317	236	25853.5	9.42
SETIT_030734mg	KQL25234	283	30571.3	8.88
SETIT_022792mg	KQL16417	299	32925.8	9.79
SETIT_022957mg	KQL16419	269	29968.7	9.97
SETIT_024657mg	KQL13516	246	27445.3	8.7
SETIT_023067mg	KQL13517	247	27419.5	8.19
SETIT_024877mg	KQL14056	236	25952.5	8.45
SETIT_022982mg	KQL15405	264	28029.7	9.43
SETIT_024373mg	KQL13511	230	25891.8	9.18
SETIT_036734mg	KQK90959	311	33621	9.55
SETIT_038941mg	KQK90904	303	33081.1	7.4
SETIT_002271mg	KQL05781	319	36169.2	9.81
SETIT_002620mg	KQL03384	257	28259.5	9.98
SETIT_004556mg	KQL05443	305	33585.4	10.62
SETIT_002478mg	KQL06581	210	23185.2	8.46
SETIT_002746mg	KQL03418	238	25867.2	4.68
SETIT_002578mg	KQL07993	219	23381.6	9.47

Gene ID	Transcript ID	length	MW(Da)	pI
Gene ID	Transcript ID	length	MW(Da)	pI
SETIT_014138mg	KQL02677	307	33010	9.62
SETIT_026700mg	KQK94691	296	32543.4	9.16
SETIT_026691mg	KQK94690	299	32906.6	5.78
SETIT_027272mg	KQK93650	258	28322.5	9.98
SORBI_3008G094400	EES15957	302	33005.2	9.87
SORBI_3008G094000	EES17001	304	33253.6	9.86
SORBI_3008G094300	OQU79094	314	34586.3	10.15
SORBI_3001G377600	EER94881	329	35194.6	9.68
SORBI_3001G373600	EER92278	313	34389.5	7.58
SORBI_3002G259301	OQU89754	273	29753.3	8.97
SORBI_3009G252000	EES20039	239	25930.5	7.38
SORBI_3009G080900	KXG21564	246	27344.3	8.55
SORBI_3009G143500	EES19554	256	27273.9	9.05
SORBI_3003G038800	KXG31662	221	24259.2	8.53
SORBI_3003G038700	EES00162	213	23693.6	4.28
SORBI_3003G269300	EES01331	231	25145.5	9.14
SORBI_3003G149000	OQU86800	242	26380.7	6.94
SORBI_3003G377700	KXG33819	269	29077.2	9.58
SORBI_3003G213000	EES01039	244	27204.3	9.56
SORBI_3003G182800	KXG32676	243	26823.6	8.8
SORBI_3003G015200	EES00047	259	28389.4	9.03
SORBI_3004G157100	EES06868	336	35688.9	7.86
SORBI_3004G133500	EES06723	250	27433.6	9.8
SORBI_3004G133600	EES06724	252	27624.7	9.62
SORBI_3004G136600	EES06744	250	27204.3	9.58
SORBI_3005G123500	KXG28442	289	31500	9.35
SORBI_3007G191200	EES14183	309	33524.5	9.73
