# Supplementary material for: Genome-Wide Investigation and Characterization of SWEET Gene Family with Focus on Their Evolution and Expression during Hormone and Abiotic Stress Response in Maize
Source: Genes (Basel). 2022 Sep 20;13(10):1682. doi: 10.3390/genes13101682 (PMC9601529; doi:10.3390/genes13101682)
Supplement: Supplementary file 1 [file genes-13-01682-s001.zip › Table S5.pdf]

Table S5. Analysis and distribution of conserved motifs in maize SWEET proteins.

Name	Motif Sequence	P-value	Width
motif1	RRVIRTKSVEFMPFSLSLFLFLSAVVWFAYGLLIKDLYVA	2.2e-476	40
motif2	KKSTEGFQSVPYVVTLLSCMLWIFYGLPKVNPL	4.2e-343	33
motif3	TPNGLGCVFGAVQLVLYLVYA	2.0e-304	21
motif4	HPWAFAFGIJGNVISFLVFLSPVPTFYRI	1.3e-267	29
motif5	RVVVVGWVCVAFSVSMYAAPL	1.1e-110	21
motif6	PKKARLFTAKJLLLLNVGVFGLVLLLTLL	3.4e-072	29
motif7	PVTQMAAVPVRSCAAEAAAPAMLPNRDVVDVFVSRHSPAVH	1.3e-027	41
motif8	RKVFLJLAAEVAFVVAVAALVJLLAHTHE	1.2e-019	29
motif9	NRNPKKNGAVSEMQQ	1.0e-006	15
motif10	PSAADEHVLVNIAKL	8.0e-006	15
