# Supplementary material for: Genome-Wide Investigation and Characterization of SWEET Gene Family with Focus on Their Evolution and Expression during Hormone and Abiotic Stress Response in Maize
Source: Genes (Basel). 2022 Sep 20;13(10):1682. doi: 10.3390/genes13101682 (PMC9601529; doi:10.3390/genes13101682)
Supplement: Supplementary file 1 [file genes-13-01682-s001.zip › Table S7.pdf]

Table S7. Primers of sequences

Gene ID	Gene Name	Forward primer（5′-3′）	Reverse primer（5′-3′）
Zm00001d013367	ZmTubulin1	5'-GTGTCCTGTCCACCCACTCTCT-3'	5'-GGAACTCGTTCACATCAACGTTC-3'
Zm00001d000222	ZmSWEET1a	5'-CAGGTTCTTCTTCGGAGTTT-3'	5'-CGATAGGAGGCAGTTGAGC-3'
Zm00001d038226	ZmSWEET1b	5'-CGAGCGTATGTTTCCTCCC-3'	5'-GCATCCCTATCGTCCACCTT-3'
Zm00001d010440	ZmSWEET3a	5'-AGTGACGGGTGGGAGAACC-3'	5'-AAGGATACGATGACGCAGA-3'
Zm00001d039347	ZmSWEET3b	5'-AGGGCAACGTGGAGGAATT-3'	5'-CGGTGCGAACCGTAGGTAT-3'
Zm00001d015905	ZmSWEET4a	5'-ATGGCATCTGCTGGACTGC-3'	5'-TCTCCTGGGTGGACTTGTAG-3'
Zm00001d015914	ZmSWEET4b	5'-AGTGGAGGTGGGCTTGGTG-3'	5'-TGTGAGTGACGAGCGAGTG-3'
Zm00001d015912	ZmSWEET4c	5'-TGCTATTGGAAACCCTTCT-3'	5'-CAAACGCTTACTACTACCTCA-3'
Zm00001d044421	ZmSWEET6a	5'-GGAGTTCAAGCCGGACCCT-3'	5'-GCTGTCGGAATAGAGGAAGAA-3'
Zm00001d011299	ZmSWEET6b	5'-GCCTTCTTCGGTCTCATCC-3'	5'-TGGGCAGCTCCACATTCTT-3'
Zm00001d031647	ZmSWEET11a	5'-TGCGGACTCTGGCCTACTT-3'	5'-CTTGACCACCTTGACGATGA-3'
Zm00001d021064	ZmSWEET11b	5'-GAGTGTAGTGGCGTAGGAGC-3'	5'-CGACAGTAACACGCACAGG-3'
Zm00001d029135	ZmSWEET12a	5'-TGGCCTGAACTAGCGAAAC-3'	5'-GCAAGGGTGACCAAGAATG-3'
Zm00001d047487	ZmSWEET12b	5'-GCGAGTGATGAGAACCAAGAGC-3'	5'-TGATGAGGAGGCCGTAGAAGAA-3'
Zm00001d023677	ZmSWEET13a	5'-TCATCACCCTAATCCAAGC-3'	5'-AGTAGACCGAAAGCAAACG-3'
Zm00001d023673	ZmSWEET13b	5'-GTCGAGTCCGAACGCATAA-3'	5'-CAAAGCTAAGGGTTGGAATAA-3'
Zm00001d041067	ZmSWEET13c	5'-CGACCAAGAAGGGCAGGAT-3'	5'-CGGAGAAGCCGACGCAGAT-3'
Zm00001d007365	ZmSWEET14a	5'-TATACGAAAGGGATGAAGACG-3'	5'-GTGACGGGACAACAAGTAGC-3'
Zm00001d049252	ZmSWEET14b	5'-CGGTAGGTCGTAACATAACAT-3'	5'-AATAATCGCGGAGGCAAGA-3'
Zm00001d050577	ZmSWEET15a	5'-CCCATGCCCTTGGAGATAA-3'	5'-CGTTGCACGGTACGGTTCT-3'
Zm00001d016590	ZmSWEET15b	5'-GAGCGCCGTGATCTGGTTC-3'	5'-GCGGGCTCCTTGTTCCTGT-3'
Zm00001d029098	ZmSWEET16	5'-TCGGGAGCATCATCAAGTC-3'	5'-ACGTCGGTCGAACAATAGC-3'
Zm00001d040656	ZmSWEET17a	5'-GACCGAGACATCTTCCTTG-3'	5'-GAGGCGACGCTATTTCTTT-3'
Zm00001d009071	ZmSWEET17b	5'-TCTACTACGGCCTCACCAA-3'	5'-AATTAAGCAACGGATTGTG-3'
